# SGLT2 Inhibitors as a Therapeutic Option for Diabetic Nephropathy

**DOI:** 10.3390/ijms18051083

**Published:** 2017-05-18

**Authors:** Daiji Kawanami, Keiichiro Matoba, Yusuke Takeda, Yosuke Nagai, Tomoyo Akamine, Tamotsu Yokota, Kazunori Sango, Kazunori Utsunomiya

**Affiliations:** 1 Division of Diabetes, Metabolism and Endocrinology, Department of Internal Medicine, Jikei University School of Medicine, 3-25-8 Nishi-shinbashi, Minato-ku, Tokyo 105-8461, Japan; matoba@jikei.ac.jp (K.M.); ms05-takeda@jikei.ac.jp (Y.T.); y.nagai@jikei.ac.jp (Y.N.); akamine-tm@igakuken.or.jp (T.A.); yokotat@jikei.ac.jp (T.Y.); kazu-utsunomiya@jikei.ac.jp (K.U.); 2 Diabetic Neuropathy Project, Department of Sensory and Motor Systems, Tokyo Metropolitan Institute of Medical Science, 2-1-6 Kamikitazawa, Setagaya-ku, Tokyo 156-8506, Japan; sango-kz@igakuken.or.jp

**Keywords:** diabetic nephropathy, cardiovascular disease, SGLT2 inhibitors

## Abstract

Diabetic nephropathy (DN) is a major cause of end-stage renal disease (ESRD) worldwide. Glycemic and blood pressure (BP) control are important but not sufficient to attenuate the incidence and progression of DN. Sodium–glucose cotransporter (SGLT) 2 inhibitors are a new class of glucose-lowering agent suggested to exert renoprotective effects in glucose lowering-dependent and independent fashions. Experimental studies have shown that SGLT2 inhibitors attenuate DN in animal models of both type 1 diabetes (T1D) and type 2 diabetes (T2D), indicating a potential renoprotective effect beyond glucose reduction. Renoprotection by SGLT2 inhibitors has been demonstrated in T2D patients with a high cardiovascular risk in randomized controlled trials (RCTs). These favorable effects of SGLT2 inhibitors are explained by several potential mechanisms, including the attenuation of glomerular hyperfiltration, inflammation and oxidative stress. In this review article, we discuss the renoprotective effects of SGLT2 inhibitors by integrating experimental findings with the available clinical data.

## 1. Introduction

Diabetes is a worldwide growing public health problem with a risk of vascular complications. Diabetic nephropathy (DN) is considered as a major microvascular complication because it causes end-stage renal disease (ESRD) and affects mortality in diabetic patients [1]. The United Kingdom Prospective Diabetes Study (UKPDS) 35 provided evidence that intensive glycemic control can inhibit the incidence or progression of microvascular complications, including DN, in patients with type 2 diabetes (T2D) [2]. Recently, the Action in Diabetes and Vascular Disease: Preterax and Diamicron Modified Release Controlled Evaluation Post-Trial Observational Study (ADVANCE-ON) demonstrated the long-term benefits of intensive glycemic control for preventing ESRD in patients with T2D [3]. However, intensive glycemic control should be carefully performed, as patients with DN are at a high risk of developing hypoglycemia due to a reduced rate of gluconeogenesis and the low clearance rate of glucose-lowering agents. In addition, severe hypoglycemia is associated with an increased risk of cardiovascular disease (CVD) [4]. Therefore, avoiding severe hypoglycemia is important for preventing CVD in patients with T2D. To satisfy this unmet need, efforts to develop a novel therapeutic strategy against DN involving anti-diabetic agents with a low hypoglycemic risk are underway. Sodium–glucose cotransporter (SGLT) 2 inhibitors are a new class of glucose-lowering agents that can induce glucosuria, resulting in optimal glycemic control in patients with T2D [5]. Recently, empagliflozin has been shown to attenuate both DN and CVD in patients with T2D [6,7]. These findings may herald a new era in treating DN. Investigations into the pleiotropic effects of SGLT2 inhibitors beyond glucose reduction have been conducted, and the mechanisms underlying the renoprotection induced by SGLT2 inhibitors are gradually becoming clear.

## 2. Pathogenesis and Current Therapeutic Strategies of DN

Although the development of DN hinges on many factors and processes, glomerular hyperfiltration, metabolic pathways, inflammation and oxidative stress have been implicated in its pathogenesis [8]. Under diabetic conditions, afferent arterioles are dilated, and blood flow into the glomeruli is increased, which causes elevated intraglomerular pressure. These changes induce inflammation and oxidative stress in the glomeruli, leading to the excessive production of extracellular matrix (ECM), which plays a key role in the development of glomerulosclerosis. As no specific therapy against DN has yet been developed, controlling hyperglycemia, hypertension and dyslipidemia is the major therapeutic strategy for DN. However, the management of these risk factors is not sufficient to prevent DN progression. With this background, therapies utilizing antidiabetic agents with pleiotropic effects have been administered. For instance, incretin-based therapies—dipeptidyl peptidase (DPP)-4 inhibitors and glucagon-like peptide-1 (GLP-1) receptor agonists—have been widely used because they have anti-inflammatory and antioxidative stress properties beyond glucose reduction [9]. Similarly, statins have been shown to exert renoprotective effects by attenuating inflammation and oxidative stress, independent of their lipid-lowering effects [8]. SGLT2 inhibitors are a new class of antidiabetic agents expected to attenuate the major paths of the pathogenesis of DN, such as glomerular hyperfiltration, inflammation, and oxidative stress.

## 3. Renal Glucose Handling by SGLT2

The kidney plays a major role in ensuring glucose homeostasis via gluconeogenesis and the reabsorption of filtered glucose in the proximal tubules. As the kidney filters approximately 180 L of plasma each day, approximately 180 g of glucose is filtered daily in normal glucose-tolerant (NGT) individuals, with a mean day-long plasma glucose concentration of 100 mg/dL [10]. In NGT individuals, glucose cannot be detected in the urine because all filtered glucose is reabsorbed in the proximal tubule by SGLTs, which couple glucose reabsorption to sodium reabsorption. Two isoforms of SGLT are found in the kidney: SGLT1 and SGLT2. SGLT2 is located in the early (S1) proximal tubule and is responsible for the majority of glucose reabsorption (80–90% of filtered glucose) [5] as a low-affinity/high-capacity system [11]. SGLT1 is located in the distal part of the proximal tubule (S2/S3) and is responsible for the reabsorption of the remaining 10–20% of filtered glucose [10,12,13] as a high-affinity/low-capacity system [11]. Although SGLT2 is primarily expressed in the kidney, SGLT1 is detected in various tissues, including the kidney, gut, heart and lungs [10].

The maximum glucose transport capacity (tubular max for glucose, T_mG_) of the proximal tubule is approximately 375 mg/min [14], and the filtered glucose load (approximately 125 mg/min or 180 g/day) is less than the maximum renal glucose transport capacity, meaning that all of the filtered glucose is reabsorbed and returned to the circulation in NGT individuals [10]. However, under diabetic conditions, the filtered glucose load is increased to approximately 375 mg/min, and the filtered glucose in excess of the T_mG_ is subsequently excreted into the urine. The plasma glucose concentration at which the filtered glucose load reaches the T_mG_ (375 mg/min) is called the threshold. The theoretical plasma glucose threshold that corresponds to the T_mG_ (375 mg/min) is approximately 300 mg/dL. However, in healthy individuals, the plasma glucose concentration at which glucose begins to appear in the urine is approximately 180 mg/dL. This difference between the “theoretical threshold” and the “actual threshold” is explained by a nonlinear shift (splay) in the glucose reabsorption and excretion curves as the T_mG_ is approached [13,15].

## 4. Regulation of SGLT2 and Glucose Reabsorption in Diabetes

It has been reported that the T_mG_ of the kidney and the threshold for the appearance of glucose in the urine are elevated in diabetic subjects [16]. The kidney increases T_mG_ by approximately 20% under diabetic conditions [17,18]. Studies utilizing type 1 diabetes (T1D) and T2D animal models have demonstrated that renal SGLT2 expression is increased by 40–80% [19,20]. Increased SGLT2 activity is also reported in db/db mice [21]. It has been shown that human kidney tubular cells obtained from the urine of T2D patients show an increased expression and glucose transport activity compared with non-diabetic individuals [22]. The expression levels of SGLT2 under diabetic conditions remain controversial because a recent study demonstrated that the expression of SGLT2 messenger RNA (mRNA) and protein was increased in renal biopsy specimens from individuals with DN, whereas the expression levels of SGLT2 protein were not changed in db/db mice [23]. Although diabetic conditions do not uniformly increase the renal expression of SGLT2, the uptake of glucose by SGLT2 has been shown to be increased, even with unchanged expression levels [24]. During SGLT2 inhibition, the glucose load to the S2/S3 segment of the proximal tubule is enhanced, and glucose reabsorption by SGLT1 is increased, despite the renal SGLT1 protein expression being downregulated [25,26]. The decreased expression of SGLT1 is thought to be a protective response to avoid glucotoxicity [27]. The excretion of filtered glucose by SGLT2 inhibition is limited to 50–60%, which is associated with a compensatory mechanism by the enhanced glucose transport of SGLT1. A study using mice lacking both SGLT1 and SGLT2 demonstrated elevated glucose excretion compared with the deletion of SGLT2 alone [28]. The upregulation of SGLT2 and enhanced SGLT2-mediated glucose reabsorption confirm the utility of SGLT2 as a therapeutic target for diabetes.

## 5. Experimental Studies

**Figure 1:**
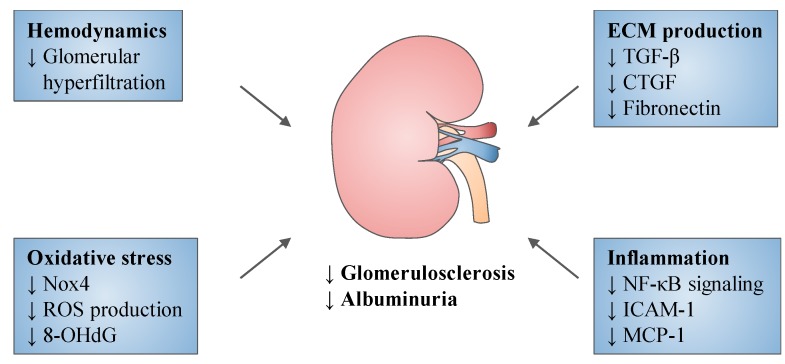
Sodium–glucose cotransporter (SGLT) 2 inhibitors exert multiple effects on diabetic nephropathy (DN). These agents improve glomerular filtration and reduce extracellular matrix (ECM) production, oxidative stress and inflammation. These effects contribute to the reduction of renal fibrosis and albuminuria. CTGF: Connective tissue growth factor; ICAM-1: Intercellular adhesion molecule-1; MCP-1: Monocyte chemoattractant protein-1; NF-κB: Nuclear factor-κB; Nox4: NADPH oxidase 4; ROS: Reactive oxygen species; TGF-β: Transforming growth factor β.

A series of studies has shown that SGLT2 inhibition exerts renoprotective benefits (Figure 1). Vallon et al. demonstrated that glomerular hyperfiltration is attenuated in SGLT2-deficient diabetic mice [20]. The administration of streptozotocin (STZ), which induces T1D in experimental animals, increased blood glucose levels to a lesser extent in SGLT2-deficient mice than in wild-type mice (approximately 300 mg/dL vs. 470 mg/dL). Interestingly, SGLT2-deficient diabetic mice showed reduced glomerular hyperfiltration compared with wild-type mice [20]. From a mechanistic standpoint, a lack of SGLT2 resulted in the decreased renal accumulation of p62, an indicator of autophagy. However, SGLT2-deficient mice did not show any inhibitory effects on albuminuria, renal growth or inflammation [20].

Notably, the pharmacologic inhibition of SGLT2 has been shown to result in stronger renoprotective effects in diabetic animals than its genetic deletion. Phlorizin, a non-specific SGLT inhibitor, has been shown to attenuate glomerular hyperfiltration, increased kidney size, and renal oxidative stress in STZ-induced diabetic rats [29,30]. Gembardt et al. showed that empagliflozin, an SGLT2 inhibitor (300 ppm in the diet for 12 weeks), ameliorates albuminuria along with inhibition of glomerular hypertrophy, mesangial expansion, and inflammatory markers in BTBR ob/ob mice with or without hypertension [31]. Vallon et al. investigated the effects of SGLT2 inhibitor empagliflozin on DN in type 1 diabetic Akita mice. Administration of empagliflozin (300 mg/kg of diet for 15 weeks) attenuated not only glomerular hyperfiltration but also albuminuria and increases in kidney weight in proportion to hyperglycemia [19]. Furthermore, empagliflozin inhibited diabetes-induced renal expression of kidney growth markers—p27, p21 and heme oxygenase (HO)-1—and inflammation markers, such as nuclear factor-κB (NF-κB) and interleukin (IL)-6 [19]. The advanced glycated end products (AGE)–receptor for AGE (RAGE) axis seems to be involved in the anti-inflammatory and antioxidative stress properties of empagliflozin. Ojima et al. showed that four-week treatment of empagliflozin (10 mg/kg) inhibited the renal expression of AGEs and RAGE and reduced the urinary excretion of 8-hydroxydeoxyguanosine (OHdG) and l-fatty acid binding protein (l-FABP), a tubular injury marker, in STZ-induced diabetic rats [32]. Although albuminuria was not prevented by empagliflozin in that study [32], the observations suggest that empagliflozin exerts renoprotective effects partly via the inhibition of the AGE–RAGE pathway. In addition to glomerular changes, empagliflozin has also been shown to inhibit high glucose-mediated inductions of collagen IV and IL-6 in proximal tubular cells [33]. Interestingly, empagliflozin did not attenuate the high glucose-induced activation of inflammatory signals, such as NF-κB, indicating that the inhibition of the fibrotic response by empagliflozin is associated with a reduction in glucotoxicity [33].

SGLT2 inhibitors other than empagliflozin have been shown to exert renoprotective effects in diabetic animal models. For instance, T2D rats (T2DN/McWi strain) that received luseogliflozin (10 mg/kg for 3 months) showed reduced glomerulosclerosis, renal fibrosis, and tubular necrosis, none of which were inhibited by insulin therapy [34], indicating that luseogliflozin exerts renoprotective effects beyond glucose reduction. With respect to proteinuria, luseogliflozin did not show any beneficial effects; however, the combination of luseogliflozin and lisinopril, an angiotensin-converting enzyme (ACE) inhibitor, potently attenuated proteinuria to a greater extent than lisinopril alone [34]. Terami et al. showed that dapagliflozin (0.1 mg/kg or 1.0 mg/kg for 12 weeks) attenuated albuminuria in a dose-dependent manner in db/db mice [35]. A pathological analysis revealed that mesangial expansion, macrophage infiltration, and tubulointerstitial fibrosis were inhibited by dapagliflozin. Hatanaka et al. reported that the administration of dapagliflozin (1.0 mg/kg for 12 weeks) to Akita mice resulted in the significant reduction of renal macrophage infiltration and interstitial fibrosis in comparison to mice treated with insulin, whereas glycemic control was equally improved in the two groups [36]. Studies using cultured proximal tubular cells have provided evidence that a reduction in the expression of inflammatory mediators—transforming growth factor-β (TGF-β), monocyte chemoattractant protein-1 (MCP-1), osteopontin, and intercellular adhesion molecule-1 (ICAM-1), oxidative stress—NADPH oxidase 4 (Nox4) expression and reactive oxygen species (ROS) production [35], and apoptosis mechanistically underlie the major actions of dapagliflozin. Nagata et al. demonstrated that the administration of diet containing 0.005% or 0.015% of tofogliflozin for eight weeks prevented the progression of albuminuria and glomerular hypertrophy in db/db mice [37]. Finally, Wang et al. showed that the treatment of db/db mice with diet containing 0.07 g/kg of the SGLT2 inhibitor JNJ39933673 for 12 weeks decreased renal inflammation and lipid accumulation by inhibiting transcriptional factors and enzymes that mediate fatty acid and triglyceride synthesis, such as carbohydrate-responsive element-binding protein (ChREBP)-β, pyruvate kinase L, stearoyl-CoA desaturase-1, and diacylglycerol *O*-acyl transferase 1 [23].

The degree of blood glucose reduction and the drug dose may be important factors for determining the renoprotective effects of SGLT2 inhibitors in animal models. Gangadharan Komala et al. reported that empagliflozin (10 mg/kg for 19 weeks) failed to attenuate the inflammatory and fibrotic responses in an endothelial nitric oxide synthase (eNOS)-deficient mouse model of STZ-induced diabetes, whereas these changes were inhibited by telmisartan, an angiotensin-II receptor blocker (ARB) [38]. Gallo et al. reported that empagliflozin (10 mg/kg for 10 weeks) attenuated the markers of renal fibrosis, such as connective tissue growth factor (CTGF), fibronectin, and TGF-β1 without improving albuminuria in db/db mice [39]. However, empagliflozin also did not attenuate the increased urinary markers of tubule damage, renal hypertrophy, or glomerulosclerosis [39]. Those authors also found that the plasma glucose level remained >15 mmol/L, whereas studies showing the inhibition of progressive albuminuria reported glucose levels <15 mmol/L. Furthermore, Gallo et al. administered a lower dose of SGLT2 inhibitors than in studies that showed potent renoprotective effects. These differences may affect the degree of renoprotective effect of SGLT2 inhibitors.

Taken together, these previous findings demonstrate the beneficial effects of SGLT2 inhibitors on DN through the attenuation of glomerular hyperfiltration as well as glomerulosclerosis and tubuleinterstitial fibrosis in multiple experimental diabetic models. SGLT2 inhibitors may have direct renoprotective effects beyond glucose lowering; however, hard conclusions remain difficult to draw, as both the glycemic and blood pressure (BP) control were improved in these experimental diabetic models. A point of interest is whether SGLT2 inhibitors have beneficial effects on non-diabetic chronic kidney disease (CKD). To date, two studies have shown that SGLT2 inhibitors failed to show renoprotective effects. Zhan et al. reported that dapagliflozin did not attenuate the progression of kidney injury in 5/6 rats that had been subjected to nephrectomy [40]. Ma et al. demonstrated that empagliflozin had no effect on an oxalate-diet-induced model of CKD that develops nephrocalcinosis-related tubular atrophy and interstitial fibrosis [41]. As such, further studies in different models will be required to confirm whether SGLT2 inhibitors exert renoprotective effects on non-diabetic kidney injury [42]. A summary of the effects of SGLT2 inhibitors on experimental DN is shown in Table 1.

## 6. Clinical Studies

The EMPA-REG OUTCOME is a clinical trial that evaluated the non-inferior cardiovascular safety of empagliflozin in high-CV-risk T2D patients with an estimated glomerular filtration rate (eGFR) of at least 30 mL/min/1.73 m^2^ [6]. In this study, patients were assigned to a placebo group, empagliflozin 10 mg daily group, or empagliflozin 25 mg daily group [6]. Approximately 80% of patients were treated with ACE inhibitors or ARBs as their standard care. The primary 3-point major adverse cardiovascular event endpoint (3P-MACE: cardiovascular death, non-fatal myocardial infarction, non-fatal stroke) was significantly reduced by 14%, and a 38% reduction in cardiovascular death was observed. Death from any cause was reduced by 32%, and hospitalization for heart failure was reduced by 35% in patients assigned to receive empagliflozin [6]. An analysis that was a prespecified component of the secondary microvascular outcome of that trial has been reported [7]. Empaglifolizn reduced the rate of new onset or worsening nephropathy, which were defined as new-onset macroalbuminuria, doubling of creatinine, and eGFR ≤ 45 mL/min/1.73 m^2^, initiation of renal replacement therapy, and death due to renal disease (hazard ratio [HR]: 0.61, 95% confidence interval [CI]: 0.53–0.70; *p* < 0.0001) [7] (Table 2). These observations support the hypothesis that empagliflozin has beneficial effects on DN. However, it is noteworthy that empagliflozin did not inhibit the new onset of microalbuminuria [7].

A secondary analysis of the Canagliflozin Treatment and Trial Analysis versus Sulphonylurea (CANTA-SU) study confirmed the renoprotective effects of canagliflozin [43]. In that study, 1450 T2D patients receiving metformin were randomly assigned to receive either once-daily canagliflozin 100 mg, canagliflozin 300 mg, or glimepiride uptitrated to 6–8 mg. Approximately 60% of patients were treated with the renin–angiotensin–aldosterone system (RAAS) inhibitors. Over two years of follow-up, the eGFR decline was significantly slower in patients receiving canagliflozin than in those receiving glimepiride (0.5 mL/min/1.73 m^2^ per year with 100 mg daily [95% CI: 0.0 to 1.0] and 0.9 mL/min/1.73 m^2^ per year with 300 mg daily [95% CI: 0.4 to 1.4], compared with glimepiride (3.3 mL/min/1.73 m^2^ per year [95% CI: 2.8 to 3.8]) [43]. In patients with albuminuria with a urinary albumin-to-creatinine ratio (ACR) ≥30 mg/g at baseline, canagliflozin 100 mg decreased the urinary ACR by 31.7% (95% CI: 8.6% to 48.9%), and canagliflozin 300 mg decreased it by 49.3% (95% CI: 31.9% to 62.2%) compared to glimepiride [43]. The HbA1c reductions at one year were 0.81% (glimepiride), 0.82% (canagliflozin 100 mg), and 0.93% (canagliflozin 300 mg), and those at two years were 0.55%, 0.65%, and 0.74%, respectively [43]. A slower eGFR decline occurred with little difference in the HbA1c levels among treatment groups [43]. These findings are consistent with the results of the EMPA-REG OUTCOME trial, indicating that SGLT2 inhibitors exert renoprotective effects beyond glucose reduction (Table 2).

Whether or not the cardio-renal protective effects of empaglifozin are a drug effect or class effect remains unclear. To clarify this point, several large RCTs utilizing canagliflozin are ongoing. The Canagliflozin and Renal Events in Diabetes with Established Nephropathy Clinical Evaluation (CRDENCE, clinical trial registration no. NCT02065791) is a randomized, double-blind, placebo-controlled, parallel-group, multicenter study of the effects of canagliflozin on the renal and cardiovascular outcomes in participants with T2D and DN, who are receiving a standard of care including a maximum tolerated daily dose of ACE inhibitors or ARBs. This study is evaluating the effects of canagliflozin on the time to the primary composite end point of ESRD, doubling of serum creatinine, or cardiovascular death. The canagliflozin cardiovascular assessment study (CANVAS, clinical trial registration no. NCT01032629) is a randomized, double-blind, placebo-controlled, parallel-group, multicenter study [44]. This study will assess the effects of canagliflozin on CVD in patients with T2D who are at a high risk of developing cardiovascular disease. The primary composite outcome is 3P-MACE (CV death, non-fatal myocardial infarction (MI), and non-fatal stroke). As a secondary outcome, the effects of canagliflozin on the progression/regression of albuminuria will be assessed. The data from this study will be combined with the data from the CANVAS-R study (Study of the Effects of Canagliflozin on Renal Endpoints in Adult Subjects with Type 2 Diabetes Mellitus, clinical trial registration no. NCT01989754). These studies will conclude whether or not renoprotection is a class effect of SGLT2 inhibitors.

## 7. Mechanisms Underlying the Renoprotection Exerted by SGLT2 Inhibitors beyond Glucose Reduction

### 7.1. BP Reduction

A meta-analysis (27 RCTs; *n* = 12,960) demonstrated that SGLT2 inhibitors significantly reduced the systolic BP (weighted mean difference: −4.0 mmHg; 95% CI: −4.4 to −3.5) and diastolic BP (weighted mean difference: −1.6 mmHg; 95% CI: −1.9 to −1.3) [45]. Although the mechanisms responsible for these BP-lowering effects remain unclear, natriuresis and reduced arterial stiffness seem to be the most significant mediators responsible for the antihypertensive effects of SGLT2 inhibitors [46]. A significant reduction in the body weight (weighted mean difference: −1.9 kg; 95% CI: −2.5 to −1.2) was observed by SGLT2 inhibitor use, but meta-regression analyses revealed that body weight reduction was not associated with BP reduction [45]. In contrast, some reports have suggested that weight loss may account for between 28% and 40% of the observed reduction in the BP [47,48]. However, the degree of contribution of SGLT2 inhibition-mediated weight loss to BP reduction remains controversial. BP reduction by SGLT2 inhibitors is associated with a reduction in the arterial stiffness, as demonstrated by the pulse wave velocity and augmentation index in patients with T1D [49]. Furthermore, markers of arterial stiffness, such as the pulse pressure, have been shown to be improved by SGLT2 inhibitors in patients with T2D [50]. These observations support the notion that improvement of arterial stiffness may be involved in BP reduction by SGLT2 inhibition.

### 7.2. Glomerular Hyperfiltration

Glomerular hyperfiltration has been suggested to be involved in the pathogenesis of DN [51]. The hemodynamic changes have been shown to be associated with neurohormones, such as RAAS [52]. In addition to such neurohormonal activation, tubuloglomerular feedback (TGF) has also been shown to be involved in the pathogenesis of DN [53]. The macula densa controls the contraction and dilatation of the afferent arterioles by sodium concentration. In response to an increased sodium concentration in the macula densa, the afferent arterioles contract to reduce the blood flow into the glomeruli. Conversely, a decreased sodium concentration in the macula densa leads to afferent arteriole dilatation to increase the blood flow into the glomeruli to maintain a constant glomerular filtration rate (GFR) [1,54]. As mentioned above, hyperglycemia results in an increase in the SGLT2 expression, leading to the increased reabsorption of glucose and sodium in the proximal tubule. Consequently, the delivery of sodium to the macula densa is decreased, with a reduction in the adenosine triphosphate (ATP) breakdown and adenosine production [55]. As adenosine is a potent vasoconstrictor, reduced adenosine activity causes afferent arteriolar vasodilatation, leading to glomerular hyperfiltration [55].

Skrtic et al. investigated the mechanisms by which empagliflozin attenuates glomerular hyperfiltration [56]. They revealed that empagliflozin reduced the renal blood flow and renal vascular resistance and enhanced the glucosuric responses, likely reflecting an increase in the afferent arteriolar tone because of an increase in the distal tubular solute delivery in T1D patients [56]. Of note, no decreases in the renal blood flow or renal vascular resistance were noted in patients with a normal GFR at baseline [56].

Similar observations have been made in T1D patients with glomerular hyperfiltration under clamped euglycemic and hyperglycemic conditions. Cherney et al. showed that empagliflozin inhibits glomerular hyperfiltration in patients with T1D [54]. They examined T1D patients with HbA1clevels of 6.5–11.0%, normal blood pressure not on RAAS inhibitors, and an eGFR of at least 60 mL/min/l.73 m^2^. Treatment with empagliflozin (25 mg) daily for eight weeks decreased the GFR, as measured by the inulin clearance under clamped euglycemic conditions, from 172 ± 23 to 139 ± 25 mL/min/1.73 m^2^ in patients with glomerular hyperfiltration at baseline. The improvement of glomerular hyperfiltration by empagliflozin under clamped euglycemic conditions is interesting. Given that the disruption of TGF occurs during hyperglycemia, this observation raises the possibility that empagliflozin improves glomerular hyperfiltration independently of the recovery of TGF during clamped euglycemia. As mentioned above, neurohormonal factors are involved in regulating glomerular hemodynamics. For instance, Cherney et al. demonstrated that cyclooxygenase (COX)-2 inhibitor reduced the GFR and prostaglandin E metabolite levels in T1D subjects with hyperfiltration during clamped euglycemia [57]. Further studies will be required to assess whether SGLT2 inhibitors can modulate the factors that affect glomerular hemodynamics, such as prostanoids. Interestingly, an acute eGFR dip was observed in both EMPA-REG OUTCOME and CANTA-SU. After this initial dip, the eGFR tended to return toward the baseline over several weeks, remaining stable thereafter. These observations suggest that attenuation of glomerular hyperfiltration in the initial phase contributed to the long-term stability of the GFR, thereby resulting in renoprotection. It remains unclear whether individuals who had an acute initial eGFR dip showed better renal outcomes in comparison to those who did not in these studies. Ito et al. investigated the effect of ipragliflozin (50 mg daily for 24 weeks) on DN in T2D patients. They demonstrated that ipragliflozin significantly reduced albuminuria and the eGFR in patients with an eGFR of ≥90 mL/min/1.73 m^2^, whereas these effects were not observed in patients with an eGFR of <90 mL/min/1.73 m^2^ [58]. These findings demonstrate the possibility that patients with glomerular hyperfiltration are vulnerable individuals who may benefit from SGLT2 inhibitors. Further studies will be required to identify patients who are suitable for SGLT2 inhibitor treatment because the albuminuria response to dapagliflozin varies between patients [59].

### 7.3. Erythropoietin

A meta-analysis showed that SGLT2 inhibitor use resulted in a significant increase in the hematocrit (%) from baseline (weighted mean difference: 2.4%; 95% CI: 2.2% to 2.6%) [45]. This observation may have been due to the stimulatory effect of SGLT2 inhibitors on the renal production of erythropoietin (EPO) [60]. EPO is an essential glycoprotein that accelerates red blood cell maturation from erythroid progenitors and facilitates erythropoiesis [61]. Under diabetic conditions, a large amount of oxygen is required in the proximal tubules due to excessive glucose reabsorption, leading to tubulointerstitial hypoxia. This causes the impairment of EPO production from the neural crest-derived fibroblasts surrounding the renal tubules [62]. These alterations are improved by SGLT2 inhibitors. Consequently, the hematocrit and oxygen supply are increased, which is beneficial for organ protection.

Recent basic and clinical studies have shown that EPO has direct renoprotective effects beyond correcting anemia. For instance, EPO has been shown to prevent podocyte injury-induced nephrotic syndrome [63,64] and improve the endothelial function in 5/6 nephrectomized rats [65]. Interestingly, Eren et al. found that EPO attenuated albuminuria and reduced tubular injury, inflammation, and interstitial fibrosis by inhibiting inflammatory markers and oxidative stress [66]. Furthermore, a clinical study showed that erythropoiesis-stimulating agents (ESA) reduced the eGFR decline in patients with CKD [67]. These findings indicate that EPO has direct renoprotective effects.

### 7.4. RAAS

Systemic and local RAAS activation have been implicated in the pathogenesis of DN [68]. SGLT2 inhibitors reduce the circulatory volume, resulting in an increase in the RAAS [69]. Although the significance of SGLT2 inhibitor-induced RAAS activation has not been clarified, it seems to be inconsistent with SGLT2 inhibitors’ capability of renoprotection, because type-1 angiotensin-II receptor (AT_1_R) activation plays an important role in the development of DN. However, RAAS activation during ACE inhibition or angiotensin-receptor blockade may favor the renoprotective effects of the RAAS cascade, including angiotensin 1–7 (Ang-1–7) production and the activation of type-2-angiotensin-II receptor (AT_2_R) and Mas receptors (MasR) [69]. In contrast to the pressor arm of the RAAS (angiotensin-II–AT_1_R axis), a significant body of evidence has shown that the depressor arm (Ang-1–7–MasR) axis exerts beneficial effects against DN [70]. Ang-1–7, which is produced from angiotensin-II by ACE2, is a heptapeptide found in the heart and kidneys and a specific ligand for MasR that has anti-inflammatory, antioxidative stress, and vasodilatory effects [71]. The renal expression of ACE2 and MasR is decreased under diabetic conditions, which are improved by Ang-1–7 [72].

These favorable effects of SGLT2 inhibitor-mediated activation of the depressor arm of the RAAS axis may occur only in combination with RAAS inhibition by ACE inhibitors and ARBs [69]. For instance, in the presence of ARBs in combination with SGLT2 inhibitors, angiotensin-II levels are increased but cannot bind to AT_1_R, consequently binding to AT_2_R. In the presence of ACE inhibitors, the ACE2/ACE ratio is increased. Therefore, SGLT2 inhibitor-mediated RAAS activation promotes Ang-1–7 production from angiotensin-II by ACE2. Further evidence will be required to elucidate the beneficial effects of RAAS activation by SGLT2 inhibitors. Clinically, empagliflozin and dapagliflozin have been shown to increase rather than suppress the plasma levels of aldosterone and angiotensin-II in patients with T1D [54] and T2D [60]. This fact may support the RAAS-mediated organ protection by SGLT2 inhibitors in the presence of ACE inhibitors or ARBs.

### 7.5. Uric Acid Levels

Increased plasma uric acid levels are associated with DN [73]. The mechanisms by which uric acid induces DN remain unclear, but endothelial dysfunction, RAAS-mediated afferent arteriolopathy, and tubulointerstitial fibrosis are thought to be involved [74]. SGLT2 inhibitors have been shown to reduce plasma uric acid levels (10–15%) as a result of increased glycosuria, leading to the secretion of uric acid in exchange for glucose reabsorption via the glucose transporter (GLUT) 9 [46,75]. As uric acid-lowering therapy has been shown to prevent kidney function loss in diabetic patients [76], SGLT2 inhibitor-mediated uric acid reduction may be beneficial, although the clinical relevance of this effect remains to be elucidated.

## 8. Conclusions and Perspectives

Recent advances in developing novel anti-diabetic agents have facilitated optimum glycemic control; however, DN has not yet been cured, indicating the need for cause-specific treatments. Although the mechanisms underlying their renoprotective effects remain to be elucidated, SGLT2 inhibitors may be an attractive therapeutic option for DN. SGLT2 inhibitor-mediated renoprotective effects are likely largely mediated by hemodynamic mechanisms. As mentioned above, combination administration with RAAS inhibitors is required to induce the pleiotropic effects of SGLT2 inhibitors, indicating that the introduction of SGLT2 inhibitors does not negate the need for conventional risk management.

Incretin-based therapies, such as DPP-4 inhibitors and GLP-1 receptor agonists, are also known to exert renoprotective effects beyond glucose reduction. These effects are associated with protection against inflammation, oxidative stress, and the fibrotic process [9]. The combination of these agents with SGLT2 inhibitors is expected to provide additive effects not only in glycemic control but also in renoprotection in patients with T2D. Further clinical studies will be needed to prove this concept. Whether or not monotherapy of SGLT2 inhibitors can attenuate DN is unclear. In the EMPA-REG OUTCOME and CANTA-SU, approximately 60–80% of participants were receiving RAAS inhibitors as part of their standard care. The results of these studies have reconfirmed the importance of the conventional management of risk factors. Novel therapeutic strategies against DN using SGLT2 inhibitors that take this point into account should be established.

## Figures and Tables

**Table 1:** Effects of Sodium–glucose cotransporter 2 (SGLT2) inhibitors (including phlorizin) on diabetic nephropathy (DN) in experimental models. SGLT2 inhibitors have been shown to attenuate DN in both type 1 diabetes (T1D) and type 2 diabetes (T2D) animal models. High-dose but not lose-dose SGLT2 inhibitors ameliorate albuminuria, but renal inflammation, oxidative stress, and fibrotic gene expression are inhibited regardless of the dose.

Study	Model	Drug/Dose/Duration	Major Effects
Malatiali et al. [29]	STZ-diabetic rats	phlorizin, 800 mg/kg, 6 days (initial dose: 400 mg/kg)	↓ Glomerular hyperfiltration,
			↓ Kidney size,
			↓ Oxidative stress
Osorio et al. [30]	STZ-diabetic rats	phlorizin, 400 mg/kg, 30 days	↓ Oxidative stress
Vallon et al. [27]	Akita mice	empagliflozin, 300 mg/kg, 15 weeks	↓ Glomerular hyperfiltration,
			↓ Albuminuria,
			↓ Kidney Weight,
			↓ Inflammation
Gembardt et al. [31]	BTBR ob/ob mice	empagliflozin, diet containing 300 ppm of empagliflozin, 12 weeks	↓ Albuminuria,
			↓ Glomerular hypertrophy,
			↓ Inflammation,
			↓ Mesangial matrix expansion
	BTBR ob/ob mice with hypertension (angiotensin-II infusion)	empagliflozin, 300 ppm, 12 weeks	↓ Albuminuria
Ojima et al. [32]	STZ-diabetic rats	empagliflozin, 10 mg/kg, 4 weeks	↔ Albuminuria,
			↓ AGE/RAGE,
			↓ Oxidative stress,
			↓ Inflammation,
			↓ Fibrotic gene markers,
			↓ Tubular injury
Gangadharan Komala et al. [38]	eNOS-deficient-STZ-diabetic mice	empagliflozin, 10 mg/kg, 19 weeks	↔ Albuminuria,
			↔ Glomerulosclerosis,
			↔ Interstitial fibrosis,
			↔ TGF-β,
			↔ Fibronectin,
			↔ MCP-1
Gallo et al. [39]	db/db mice	empagliflozin, 10 mg/kg, 10 weeks	↔ Albuminuria
			↓ Profibrotic gene markers,
			↓ Fibronectin,
			↓ TGF-β
Kojima et al. [34]	T2DN rats (McWi strain)	luseogliflozin, 10 mg/kg, 3 weeks	↔ Proteinuria
			↓ Glomerulosclerosis
Terami et al. [35]	db/db mice	dapagliflozin, 0.1 mg/kg or 1.0 mg/kg, 12 weeks	↓ Albuminuria,
			↓ Oxidative stress,
			↓ Inflammation
Hatanaka et al. [36]	Akita mice	dapagliflozin, 1.0 mg/kg, 12 weeks	↓ Albuminuria,
			↓ Oxidative stress,
			↓ Macrophage infiltration
Nagata et al. [37]	db/db mice	tofogliflozin, Diet containing 0.005% or 0.015% of tofogliflozin, 8 weeks	↓ Albuminuria,
			↓ Glomerular hypertrophy
Wang et al. [23]	db/db mice	JNJ39933673, Diet containing 0.07 g/kg of JNJ39933673, 12 weeks	↓ Albuminuria,
			↓ Mesangial expansion,
			↓ Podocyte injury,
			↓ Renal lipid accumulation

STZ: Streptozotocin; eNOS: Endothelial nitric oxide synthase; TGF-β: Transformative growth factor β; MCP-1: Monocyte chemoattractant protein-1; AGE–RAGE: Advanced glycated end products-receptor for AGE; T2DN: Type 2 diabetic nephropathy.

**Table 2:** Randomized controlled trials (RCTs) that evaluated the effects of SGLT2 inhibitors on DN. Empagliflozin (EMPA-REG OUTCOME) and canagliflozin (CANTA-SU) have been shown to inhibit the progression of DN in patients with T2D. In the EMPA-REG OUTCOME, the reduction in the HbA1c levels by empagliflozin was approximately 0.4% compared to the placebo agent. In the CANTA-SU, the differences in the hemoglobin (Hb)A1c levels between the groups were small, indicating that SGLT2 inhibitors exert renoprotective effects independent of their glucose-lowering effects.

Trial	Drug/Dose	Patients	Major Renal Outcome
EMPA-REG OUTCOME [6,7] (3.1 years)	empagliflozin 10 mg or 25 mg/day (vs. placebo)	T2D patients at high risk of CVD with an eGFR of at least 30 mL/min/1.73 m^2^ (*n* = 7020)	↓ Incidence or worsening of DN (HR: 0.61, 95% CI: 0.53-0.70)
			↓ Progression to macroalbuminuria (HR: 0.62, 95% CI: 0.54-0.72)
			↓ Doubling of serum creatinine level accompanied by eGFR of ≤45 mL/min/1.73 m^2^ (HR: 0.56, 95% CI: 0.39-0.79)
			↓ Initiation of renal replacement therapy (HR: 0.45, 95% CI: 0.21-0.97)
CANTA-SU [38] (2 years)	canagliflozin 100 mg or 300 mg/day (vs. glimepiride 6–8 mg/day)	T2D patients who receive metformin (*n* = 1450)	↓ eGFR decline −0.5 (canagliflozin 100 mg), −0.9 (canagliflozin 300 mg), -3.3 (glimepiride) mL/min/1.73 m^2^ at 2 years
			↓ Albuminuria −31.7% (canagliflozin 100 mg), −49.3% (canagliflozin 300 mg) relative to glimepiride

HR: Hazard ratio; CI: Confidence interval.

## References

[1] De Nicola L., Gabbai F.B., Liberti M.E., Sagliocca A., Conte G., Minutolo R. (2014). Sodium/glucose cotransporter 2 inhibitors and prevention of diabetic nephropathy: Targeting the renal tubule in diabetes. Am. J. Kidney Dis..

[2] Stratton I.M., Adler A.I., Neil H.A., Matthews D.R., Manley S.E., Cull C.A., Hadden D., Turner R.C., Holman R.R. (2000). Association of glycaemia with macrovascular and microvascular complications of type 2 diabetes (UKPDS 35): Prospective observational study. BMJ.

[3] Wong M.G., Perkovic V., Chalmers J., Woodward M., Li Q., Cooper M.E., Hamet P., Harrap S., Heller S., MacMahon S. (2016). Long-term benefits of intensive glucose control for preventing end-stage kidney disease: ADVANCE-ON. Diabetes Care.

[4] Goto A., Arah O.A., Goto M., Terauchi Y., Noda M. (2013). Severe hypoglycaemia and cardiovascular disease: Systematic review and meta-analysis with bias analysis. BMJ.

[5] Abdul-Ghani M.A., DeFronzo R.A. (2014). Lowering plasma glucose concentration by inhibiting renal sodium-glucose cotransport. J. Intern. Med..

[6] Zinman B., Wanner C., Lachin J.M., Fitchett D., Bluhmki E., Hantel S., Mattheus M., Devins T., Johansen O.E., Woerle H.J. (2015). Empagliflozin, cardiovascular outcomes, and mortality in type 2 diabetes. N. Engl. J. Med..

[7] Wanner C., Inzucchi S.E., Lachin J.M., Fitchett D., von Eynatten M., Mattheus M., Johansen O.E., Woerle H.J., Broedl U.C., Zinman B. (2016). Empagliflozin and progression of kidney disease in type 2 diabetes. N. Engl. J. Med..

[8] Kawanami D., Matoba K., Utsunomiya K. (2016). Signaling pathways in diabetic nephropathy. Histol. Histopathol..

[9] Kawanami D., Matoba K., Sango K., Utsunomiya K. (2016). Incretin-based therapies for diabetic complications: Basic mechanisms and clinical evidence. Int. J. Mol. Sci..

[10] Abdul-Ghani M.A., Norton L., DeFronzo R.A. (2015). Renal sodium-glucose cotransporter inhibition in the management of type 2 diabetes mellitus. Am. J. Physiol. Ren. Physiol..

[11] Zanoli L., Granata A., Lentini P., Rastelli S., Fatuzzo P., Rapisarda F., Castellino P. (2015). Sodium-glucose linked transporter-2 inhibitors in chronic kidney disease. Sci. World J..

[12] Vrhovac I., Balen Eror D., Klessen D., Burger C., Breljak D., Kraus O., Radovic N., Jadrijevic S., Aleksic I., Walles T. (2015). Localizations of Na^+^-d-glucose cotransporters SGLT1 and SGLT2 in human kidney and of SGLT1 in human small intestine, liver, lung, and heart. Pflug. Arch..

[13] Wright E.M., Loo D.D., Hirayama B.A. (2011). Biology of human sodium glucose transporters. Physiol. Rev..

[14] Farber S.J., Berger E.Y., Earle D.P. (1951). Effect of diabetes and insulin of the maximum capacity of the renal tubules to reabsorb glucose. J. Clin. Investig..

[15] Abdul-Ghani M.A., Norton L., Defronzo R.A. (2011). Role of sodium-glucose cotransporter 2 (SGLT 2) inhibitors in the treatment of type 2 diabetes. Endocr. Rev..

[16] DeFronzo R.A., Norton L., Abdul-Ghani M. (2017). Renal, metabolic and cardiovascular considerations of SGLT2 inhibition. Nat. Rev. Nephrol..

[17] Mogensen C.E. (1971). Maximum tubular reabsorption capacity for glucose and renal hemodynamcis during rapid hypertonic glucose infusion in normal and diabetic subjects. Scand. J. Clin. Lab. Investig..

[18] Novikov A., Vallon V. (2016). Sodium glucose cotransporter 2 inhibition in the diabetic kidney: An update. Curr. Opin. Nephrol. Hypertens..

[19] Vallon V., Gerasimova M., Rose M.A., Masuda T., Satriano J., Mayoux E., Koepsell H., Thomson S.C., Rieg T. (2014). SGLT2 inhibitor empagliflozin reduces renal growth and albuminuria in proportion to hyperglycemia and prevents glomerular hyperfiltration in diabetic Akita mice. Am. J. Physiol. Ren. Physiol..

[20] Vallon V., Rose M., Gerasimova M., Satriano J., Platt K.A., Koepsell H., Cunard R., Sharma K., Thomson S.C., Rieg T. (2013). Knockout of Na-glucose transporter SGLT2 attenuates hyperglycemia and glomerular hyperfiltration but not kidney growth or injury in diabetes mellitus. Am. J. Physiol. Ren. Physiol..

[21] Arakawa K., Ishihara T., Oku A., Nawano M., Ueta K., Kitamura K., Matsumoto M., Saito A. (2001). Improved diabetic syndrome in C57BL/KsJ-db/db mice by oral administration of the Na^+^-glucose cotransporter inhibitor T-1095. Br. J. Pharmacol..

[22] Rahmoune H., Thompson P.W., Ward J.M., Smith C.D., Hong G., Brown J. (2005). Glucose transporters in human renal proximal tubular cells isolated from the urine of patients with non-insulin-dependent diabetes. Diabetes.

[23] Wang X.X., Levi J., Luo Y., Myakala K., Herman-Edelstein M., Qiu L., Wang D., Peng Y., Grenz A., Lucia S. (2017). SGLT2 protein expression is increased in human diabetic nephropathy: SGLT2 protein inhibition decreases renal lipic accumulation, inflammation, and the development of nephropathy in diabetic mice. J. Biol. Chem..

[24] Vallon V., Thomson S.C. (2012). Renal function in diabetic disease models: The tubular system in the pathophysiology of the diabetic kidney. Annu. Rev. Physiol..

[25] Vallon V., Platt K.A., Cunard R., Schroth J., Whaley J., Thomson S.C., Koepsell H., Rieg T. (2011). SGLT2 mediates glucose reabsorption in the early proximal tubule. J. Am. Soc. Nephrol..

[26] Rieg T., Masuda T., Gerasimova M., Mayoux E., Platt K., Powell D.R., Thomson S.C., Koepsell H., Vallon V. (2014). Increase in SGLT1-mediated transport explains renal glucose reabsorption during genetic and pharmacological SGLT2 inhibition in euglycemia. Am. J. Physiol. Ren. Physiol..

[27] Vallon V. (2015). The mechanisms and therapeutic potential of SGLT2 inhibitors in diabetes mellitus. Annu. Rev. Med..

[28] Powell D.R., DaCosta C.M., Gay J., Ding Z.M., Smith M., Greer J., Doree D., Jeter-Jones S., Mseeh F., Rodriguez L.A. (2013). Improved glycemic control in mice lacking Sglt1 and Sglt2. Am. J. Physiol. Endocrinol. Metab..

[29] Malatiali S., Francis I., Barac-Nieto M. (2008). Phlorizin prevents glomerular hyperfiltration but not hypertrophy in diabetic rats. Exp. Diabetes Res..

[30] Osorio H., Coronel I., Arellano A., Pacheco U., Bautista R., Franco M., Escalante B. (2012). Sodium-glucose cotransporter inhibition prevents oxidative stress in the kidney of diabetic rats. Oxid. Med. Cell. Longev..

[31] Gembardt F., Bartaun C., Jarzebska N., Mayoux E., Todorov V.T., Hohenstein B., Hugo C. (2014). The SGLT2 inhibitor empagliflozin ameliorates early features of diabetic nephropathy in BTBR ob/ob type 2 diabetic mice with and without hypertension. Am. J. Physiol. Ren. Physiol..

[32] Ojima A., Matsui T., Nishino Y., Nakamura N., Yamagishi S. (2015). Empagliflozin, an inhibitor of sodium-glucose cotransporter 2 exerts anti-inflammatory and antifibrotic effects on experimental diabetic nephropathy partly by suppressing AGEs-receptor axis. Horm. Metab. Res..

[33] Panchapakesan U., Pegg K., Gross S., Komala M.G., Mudaliar H., Forbes J., Pollock C., Mather A. (2013). Effects of SGLT2 inhibition in human kidney proximal tubular cells—Renoprotection in diabetic nephropathy?. PLoS ONE.

[34] Kojima N., Williams J.M., Takahashi T., Miyata N., Roman R.J. (2013). Effects of a new SGLT2 inhibitor, luseogliflozin, on diabetic nephropathy in T2DN rats. J. Pharmacol. Exp. Ther..

[35] Terami N., Ogawa D., Tachibana H., Hatanaka T., Wada J., Nakatsuka A., Eguchi J., Horiguchi C.S., Nishii N., Yamada H. (2014). Long-term treatment with the sodium glucose cotransporter 2 inhibitor, dapagliflozin, ameliorates glucose homeostasis and diabetic nephropathy in db/db mice. PLoS ONE.

[36] Hatanaka T., Ogawa D., Tachibana H., Eguchi J., Inoue T., Yamada H., Takei K., Makino H., Wada J. (2016). Inhibition of SGLT2 alleviates diabetic nephropathy by suppressing high glucose-induced oxidative stress in type 1 diabetic mice. Pharmacol. Res. Perspect..

[37] Nagata T., Fukuzawa T., Takeda M., Fukazawa M., Mori T., Nihei T., Honda K., Suzuki Y., Kawabe Y. (2013). Tofogliflozin, a novel sodium-glucose co-transporter 2 inhibitor, improves renal and pancreatic function in db/db mice. Br. J. Pharmacol..

[38] Gangadharan Komala M., Gross S., Mudaliar H., Huang C., Pegg K., Mather A., Shen S., Pollock C.A., Panchapakesan U. (2014). Inhibition of kidney proximal tubular glucose reabsorption does not prevent against diabetic nephropathy in type 1 diabetic eNOS knockout mice. PLoS ONE.

[39] Gallo L.A., Ward M.S., Fotheringham A.K., Zhuang A., Borg D.J., Flemming N.B., Harvie B.M., Kinneally T.L., Yeh S.M., McCarthy D.A. (2016). Once daily administration of the SGLT2 inhibitor, empagliflozin, attenuates markers of renal fibrosis without improving albuminuria in diabetic db/db mice. Sci. Rep..

[40] Zhang Y., Thai K., Kepecs D.M., Gilbert R.E. (2016). Sodium-glucose linked cotransporter-2 inhibition does not attenuate disease progression in the rat remnant kidney model of chronic kidney disease. PLoS ONE.

[41] Ma Q., Steiger S., Anders H.J. (2017). Sodium glucose transporter-2 inhibition has no renoprotective effects on non-diabetic chronic kidney disease. Physiol. Rep..

[42] Gilbert R.E. (2014). Sodium-glucose linked transporter-2 inhibitors: Potential for renoprotection beyond blood glucose lowering?. Kidney Int..

[43] Heerspink H.J., Desai M., Jardine M., Balis D., Meininger G., Perkovic V. (2016). Canagliflozin slows progression of renal function decline independently of glycemic effects. J. Am. Soc. Nephrol..

[44] Neal B., Perkovic V., de Zeeuw D., Mahaffey K.W., Fulcher G., Stein P., Desai M., Shaw W., Jiang J., Vercruysse F. (2013). Rationale, design, and baseline characteristics of the Canagliflozin Cardiovascular Assessment Study (CANVAS)—A randomized placebo-controlled trial. Am. Heart J..

[45] Baker W.L., Smyth L.R., Riche D.M., Bourret E.M., Chamberlin K.W., White W.B. (2014). Effects of sodium-glucose co-transporter 2 inhibitors on blood pressure: A systematic review and meta-analysis. J. Am. Soc. Hypertens..

[46] Heerspink H.J., Perkins B.A., Fitchett D.H., Husain M., Cherney D.Z. (2016). Sodium glucose cotransporter 2 inhibitors in the treatment of diabetes mellitus: Cardiovascular and kidney effects, potential mechanisms, and clinical applications. Circulation.

[47] Sjostrom C.D., Hashemi M., Sugg J., Ptaszynska A., Johnsson E. (2015). Dapagliflozin-induced weight loss affects 24-week glycated haemoglobin and blood pressure levels. Diabetes Obes. Metab..

[48] Cefalu W.T., Stenlof K., Leiter L.A., Wilding J.P., Blonde L., Polidori D., Xie J., Sullivan D., Usiskin K., Canovatchel W. (2015). Effects of canagliflozin on body weight and relationship to HbA_1c_ and blood pressure changes in patients with type 2 diabetes. Diabetologia.

[49] Cherney D.Z., Perkins B.A., Soleymanlou N., Har R., Fagan N., Johansen O.E., Woerle H.J., von Eynatten M., Broedl U.C. (2014). The effect of empagliflozin on arterial stiffness and heart rate variability in subjects with uncomplicated type 1 diabetes mellitus. Cardiovasc. Diabetol..

[50] Chilton R., Tikkanen I., Cannon C.P., Crowe S., Woerle H.J., Broedl U.C., Johansen O.E. (2015). Effects of empagliflozin on blood pressure and markers of arterial stiffness and vascular resistance in patients with type 2 diabetes. Diabetes Obes. Metab..

[51] Anderson S., Brenner B.M. (1988). Pathogenesis of diabetic glomerulopathy: Hemodynamic considerations. Diabetes Metab. Rev..

[52] Sochett E.B., Cherney D.Z., Curtis J.R., Dekker M.G., Scholey J.W., Miller J.A. (2006). Impact of renin angiotensin system modulation on the hyperfiltration state in type 1 diabetes. J. Am. Soc. Nephrol..

[53] Thomson S.C., Vallon V., Blantz R.C. (2004). Kidney function in early diabetes: The tubular hypothesis of glomerular filtration. Am. J. Physiol. Ren. Physiol..

[54] Cherney D.Z., Perkins B.A., Soleymanlou N., Maione M., Lai V., Lee A., Fagan N.M., Woerle H.J., Johansen O.E., Broedl U.C. (2014). Renal hemodynamic effect of sodium-glucose cotransporter 2 inhibition in patients with type 1 diabetes mellitus. Circulation.

[55] Schnermann J., Levine D.Z. (2003). Paracrine factors in tubuloglomerular feedback: Adenosine, ATP, and nitric oxide. Annu. Rev. Physiol..

[56] Skrtic M., Yang G.K., Perkins B.A., Soleymanlou N., Lytvyn Y., von Eynatten M., Woerle H.J., Johansen O.E., Broedl U.C., Hach T. (2014). Characterisation of glomerular haemodynamic responses to SGLT2 inhibition in patients with type 1 diabetes and renal hyperfiltration. Diabetologia.

[57] Cherney D.Z., Miller J.A., Scholey J.W., Bradley T.J., Slorach C., Curtis J.R., Dekker M.G., Nasrallah R., Hebert R.L., Sochett E.B. (2008). The effect of cyclooxygenase-2 inhibition on renal hemodynamic function in humans with type 1 diabetes. Diabetes.

[58] Ito D., Ikuma-Suwa E., Inoue K., Kaneko K., Yanagisawa M., Inukai K., Noda M., Shimada A. (2017). Effects of ipragliflozin on diabetic nephropathy and blood pressure in patients with type 2 diabetes: An open-label study. J. Clin. Med. Res..

[59] Petrykiv S.I., Laverman G.D., Zeeuw D., Heerspink H.J. (2017). The albuminuria lowering response to dapagliflozin is variable and reproducible between individual patients. Diabetes Obes. Metab..

[60] Lambers Heerspink H.J., de Zeeuw D., Wie L., Leslie B., List J. (2013). Dapagliflozin a glucose-regulating drug with diuretic properties in subjects with type 2 diabetes. Diabetes Obes. Metab..

[61] Tanaka T., Nangaku M. (2012). Recent advances and clinical application of erythropoietin and erythropoiesis-stimulating agents. Exp. Cell Res..

[62] Sano M., Takei M., Shiraishi Y., Suzuki Y. (2016). Increased hematocrit during sodium-glucose cotransporter 2 inhibitor therapy indicates recovery of tubulointerstitial function in diabetic kidneys. J. Clin. Med. Res..

[63] Eto N., Wada T., Inagi R., Takano H., Shimizu A., Kato H., Kurihara H., Kawachi H., Shankland S.J., Fujita T. (2007). Podocyte protection by darbepoetin: Preservation of the cytoskeleton and nephrin expression. Kidney Int..

[64] Aizawa K., Takeda S., Tashiro Y., Yorozu K., Hirata M., Kanada H., Moriguchi Y., Endo K. (2012). Renoprotection by continuous erythropoietin receptor activator in puromycin aminonucleoside-induced nephrotic syndrome. Am. J. Nephrol..

[65] Serizawa K., Yogo K., Tashiro Y., Aizawa K., Kawasaki R., Hirata M., Endo K. (2015). Epoetin β pegol prevents endothelial dysfunction as evaluated by flow-mediated dilation in chronic kidney disease rats. Eur. J. Pharmacol..

[66] Eren Z., Gunal M.Y., Ari E., Coban J., Cakalagaoglu F., Caglayan B., Beker M.C., Akdeniz T., Yanikkaya G., Kilic E. (2016). Pleiotropic and renoprotective effects of erythropoietin β on experimental diabetic nephropathy model. Nephron.

[67] Tsuruya K., Yoshida H., Suehiro T., Fujisaki K., Masutani K., Kitazono T. (2016). Erythropoiesis-stimulating agent slows the progression of chronic kidney disease: A possibility of a direct action of erythropoietin. Ren. Fail..

[68] Koitka A., Tikellis C. (2008). Advances in the renin-angiotensin-aldosterone system: Relevance to diabetic nephropathy. Sci. World J..

[69] Muskiet M.H., van Raalte D.H., van Bommel E.J., Smits M.M., Tonneijck L. (2015). Understanding EMPA-REG OUTCOME. Lancet Diabetes Endocrinol..

[70] Padda R.S., Shi Y., Lo C.S., Zhang S.L., Chan J.S. (2015). Angiotensin-(1–7): A Novel peptide to treat hypertension and nephropathy in diabetes?. J. Diabetes Metab..

[71] Prestes T.R., Rocha N.P., Miranda A.S., Teixeira A.L., Simoes-E-Silva A.C. (2016). The anti-inflammatory potential of ACE2/Angiotensin-(1–7)/Mas receptor axis: Evidence from basic and clinical research. Curr. Drug Targets.

[72] Shi Y., Lo C.S., Padda R., Abdo S., Chenier I., Filep J.G., Ingelfinger J.R., Zhang S.L., Chan J.S. (2015). Angiotensin-(1–7) prevents systemic hypertension, attenuates oxidative stress and tubulointerstitial fibrosis, and normalizes renal angiotensin-converting enzyme 2 and Mas receptor expression in diabetic mice. Clin. Sci..

[73] Bjornstad P., Lanaspa M.A., Ishimoto T., Kosugi T., Kume S., Jalal D., Maahs D.M., Snell-Bergeon J.K., Johnson R.J., Nakagawa T. (2015). Fructose and uric acid in diabetic nephropathy. Diabetologia.

[74] Jalal D.I., Maahs D.M., Hovind P., Nakagawa T. (2011). Uric acid as a mediator of diabetic nephropathy. Semin. Nephrol..

[75] Lytvyn Y., Skrtic M., Yang G.K., Yip P.M., Perkins B.A., Cherney D.Z. (2015). Glycosuria-mediated urinary uric acid excretion in patients with uncomplicated type 1 diabetes mellitus. Am. J. Physiol. Ren. Physiol..

[76] Maahs D.M., Caramori L., Cherney D.Z., Galecki A.T., Gao C., Jalal D., Perkins B.A., Pop-Busui R., Rossing P., Mauer M. (2013). Uric acid lowering to prevent kidney function loss in diabetes: The preventing early renal function loss (PERL) allopurinol study. Curr. Diab. Rep..

